# Circular RNAs and Cardiovascular Regeneration

**DOI:** 10.3389/fcvm.2021.672600

**Published:** 2021-04-13

**Authors:** Ling Tang, Pengsheng Li, Michelle Jang, Wuqiang Zhu

**Affiliations:** Department of Cardiovascular Diseases, Physiology and Biomedical Engineering, Center of Regenerative Medicine, Mayo Clinic, Scottsdale, AZ, United States

**Keywords:** circular RNA, cardiovascular disease, pathogenesis, cardiomyocyte, regeneration

## Abstract

circular RNAs (circRNAs) are a type of non-coding RNAs that are widely present in eukaryotic cells. They have the characteristics of stable structure, high abundance, and cell or tissue specific expression. circRNAs are single-stranded RNAs that are covalently back spliced to form closed circular loops. They may participate in gene expression and regulation through a variety of action modes. circRNAs can encode proteins or function by acting as miRNA sponges for protein translation. Since 2016, a growing number of research studies have shown that circRNAs play important role in the pathogenesis of cardiovascular disease. With the construction of circRNA database, the differential expression of circRNAs in the heart tissue samples from different species and the gradual elucidation of its mode of action in disease may become an ideal diagnosis biomarker and an effective therapeutic target. What can be expected surely has a broader application prospect. In this review, we summarize recent publications on circRNA biogenesis, expression profiles, functions, and the most recent studies of circRNAs in the field of cardiovascular diseases with special emphasis on cardiac regeneration.

## Introduction

Circular RNAs (circRNAs) are single-stranded RNAs that, unlike linear RNA, form a covalently closed continuous loop without 5′ end caps or 3′ Poly (A) tails. The concept of “circular RNA” was introduced by Sanger et al. when the team found that viroids are single-stranded covalently closed circRNA molecules (1). The cytoplasmic localization of circular RNA in eukaryotic cells was discovered by Hsu et al. through the electron microscope in 1979 (2). These pilot studies established the foundation of this research field.

Transcription of circRNAs had been a mystery for many years. The circular transcription of the Sry gene was discovered in mice in the early 1990s (3). In 2012, Salzman et al. (4) discovered that circRNA is a transformed transcript produced by reverse splicing of mRNA precursor and found that it is abundantly present in different types of human cells. As the field advances rapidly, a large number of circRNAs were discovered with the utilization of high-throughput sequencing technology, and their biological functions were intensively investigated. In 2016, Hansen et al. (5) found that circular RNA can act as a sponge of microRNA (miRNA) to regulate the growth and development of cells. This study shed new light on the circRNA field. Most recently, Li et al. developed a quickly screening and discovering tool for functional circular RNAs based on the CRISPR-Cas13d system, and discovered a set of functionalities that are important for cell growth and embryonic development (6). This technology provided a new research tool to the circRNA field.

Cardiovascular diseases (CVDs) are the leading cause of mortality worldwide. Several lines of evidence showed that circRNAs play important roles in regulating cardiovascular function. Jakobi et al. were the first group to provide a comprehensive catalog of RNase R-resistant circRNA species for the adult murine heart and explored the circRNA landscape of heart tissue (7). Over the next years, studies had reported that circRNAs are involved in the regulation of the physiology and pathology of the cardiovascular system. In particular, it is noted that circRNAs are involved in the pathogenesis of CVDs, such as myocardial infarction (MI) (8–14), heart failure (15, 16) and coronary artery disease (CAD) (17–23). Some circRNAs served as potential biomarkers for the diagnosis of CVDs (24–26). These findings suggest that circRNAs may be the new target molecules for the diagnosis and treatment of CVDs. In this review, we summarize circRNA classification, biogenesis, properties, functions, and some new research progress in the field of CVDs.

## Classification of circRNA

circRNAs can be divided into three types according to the different sources of the sequences: ecRNAs (exonic circRNAs) which are derived from single or multiple exons (4, 27), ciRNAs (circular intronic RNAs) which are derived from introns (28), EIciRNAs (exon-intron circRNAs) which are composed of exons and introns (29) and tricRNAs (tRNA intronic circRNAs) which are formed by splicing tRNA introns (30).

## Biogenesis of circRNA

**Figure 1:**
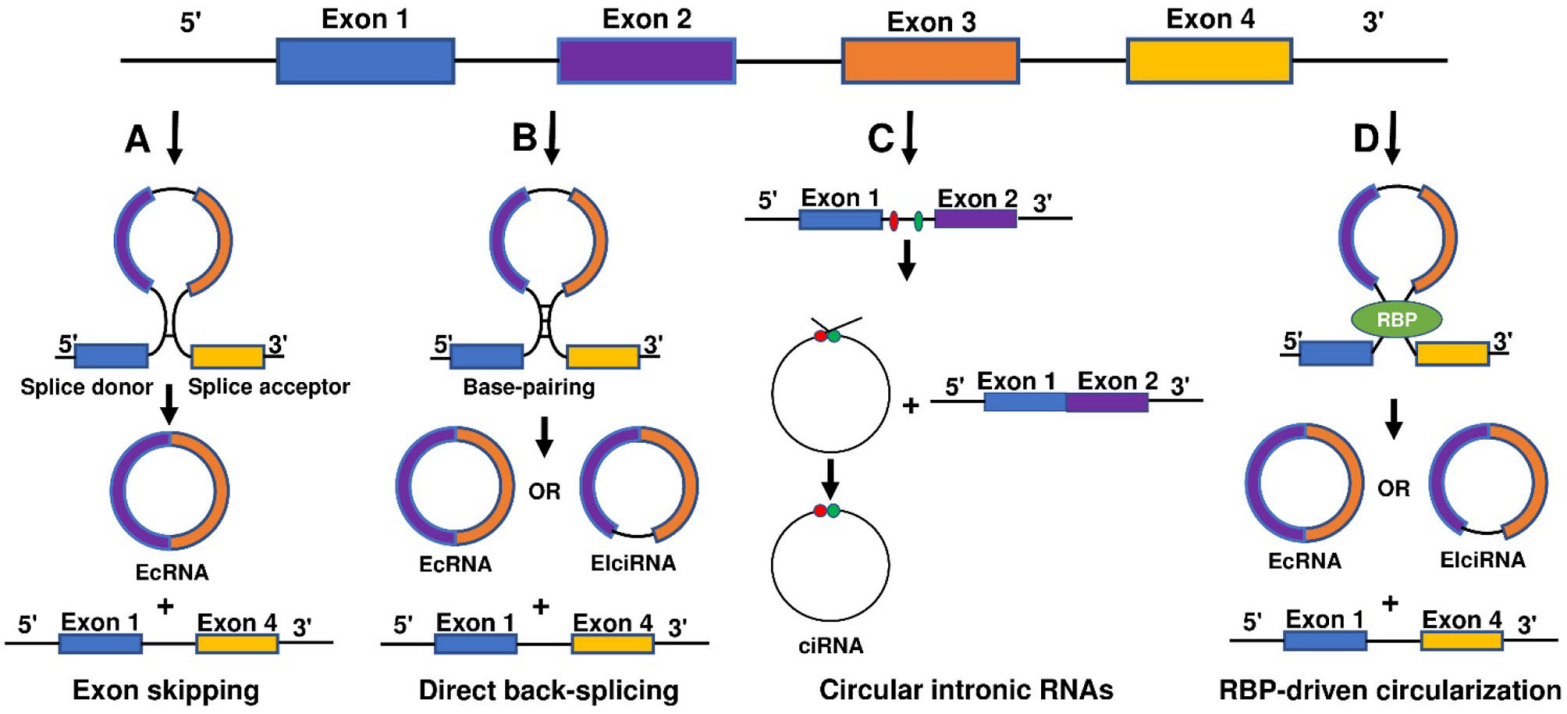
Biogenesis of circ RNAs. There are four main models for the formation of circRNA loops from pre-mRNAs including **(A)** lariat-driven circularization (exon skipping), **(B)** intron-pairing driven circularization (direct back-splicing), **(C)** circular intronic RNAs, and **(D)** RNA-binding protein (RBP)-driven circularization.

There are four primary models for the formation of circRNA loops from pre-mRNAs (Figure 1), namely lariat-driven circularization (exon skipping), intron-pairing driven circularization (direct back-splicing), circular intronic RNAs, and RNA-binding protein (RBP)-driven circularization.

Lariat-driven circularization (exon skipping) is formed by connecting the splice site of 30 nucleotides upstream of the exon to the site of 50 nucleotides downstream (Figure 1A). This connection leads to exon-skipping and the formation of an RNA lariat consisting of several exons and introns. The introns are then removed to generate circRNAs (27, 31).

Intron-pairing driven circularization (direct back-splicing) is formed when pre-mRNA flanking introns contain inverted complementary sequences (Figure 1B). The complementary pairing on both sides of the intron can lead to alternative cyclization and then a generation of various circRNAs, including ecircRNAs and EIciRNAs (27, 32). Furthermore, longer introns can be found in the flanking sequences of circRNAs, and reverse complementary sequences in longer introns can aid the formation of circRNAs (29, 33).

Circular intronic RNAs are produced by eukaryotic spliceosome-mediated splicing (Figure 1C). The lariat intron generated from the splicing reaction evades normal debranching and degradation, and the 3′ “tail” downstream from the branchpoint is trimmed leading to the formation of a stable circRNA. Conserved motifs at both ends, including the 7-nt GU-rich element near the 5′ splice site and the 11-nt C-rich element near the branch point site, are combined to prevent introns form circular branches, which promote the formation of loop structures (28, 34).

Reverse complementary sequences, such as Alu repeats, are located in upstream and downstream introns. RBP-driven circularization is formed when certain transactivator RNA binding proteins that bind to each flanking intron trigger the splicing of the donor and acceptor sites close enough to form circRNA (Figure 1D) (32, 34–38).

## Function of circRNA

**Figure 2:**
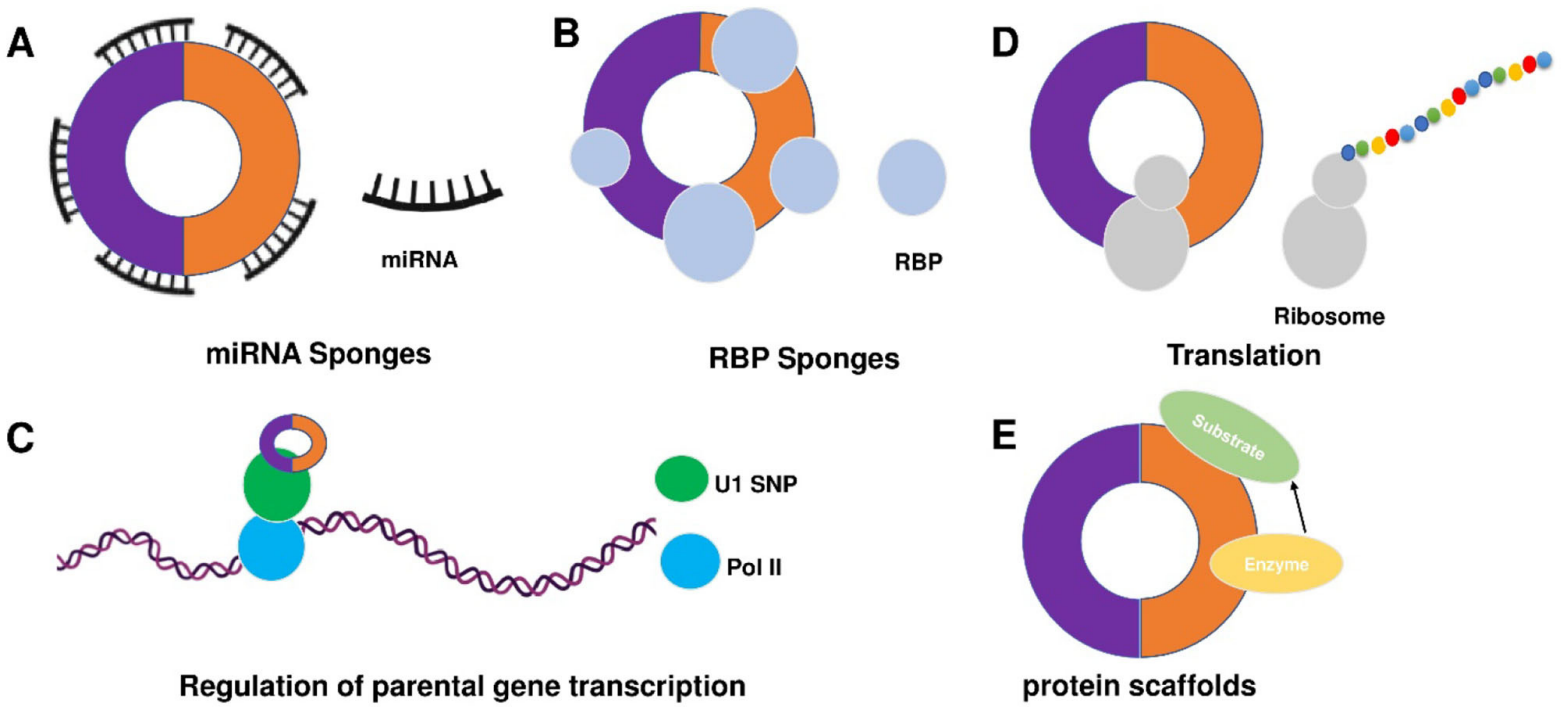
Mechanisms of circRNA functions. There are five main action mechanisms of circRNAs, including **(A)** circRNAs can act as miRNAs sponge, **(B)** circRNAs can function as RBP sponges, **(C)** circRNAs mediated regulation of the transcription of parental genes, **(D)** circRNAs can be translated into proteins via some modification, and **(E)** circRNAs can act as dynamic scaffolding molecules that modulate protein-protein interactions.

Despite the rapid growth in the field, the biological functions of circRNAs in eukaryotic cells have not been fully understood. circRNAs share some common characters. First, circRNAs are widely distributed and abundantly expressed in a diverse of cells. circRNAs can be found in a large amount in the cytoplasm of eukaryotic cells derived from animals and plants (27, 39). In humans, more than 30,000 circRNAs have been discovered and are still increasing year by year (40, 41). Second, circRNAs are stable. Due to the covalently closed structure, circRNA is resistant to degradation by ribonuclease (RNase) or exonuclease and is more stable than linear RNA (42). The expression of circRNA differs according to time, tissues, or species (39, 43). circRNA profiles change at different stages of cardiac differentiation or during cardiogenic differentiation of induced pluripotent stem cells (44, 45). Moreover, circRNAs are evolutionarily conserved (43, 46). In 2016, Werfel et al. (47) reported high homology of 1288 circRNAs across humans, mice, and rats. However, many studies have also illustrated that circRNAs are species-specific (48, 49). circRNAs show different expression profiles between normal and diseased tissues (45, 47, 50). Increasing evidence suggests some circRNAs are derived from genomic loci associated with human diseases, and contribute to transcriptional, post-transcriptional, and translational regulations (51). To summarize, there are four main modes for circRNA function (Figure 2).

**1) circRNAs can act as competitive endogenous RNAs (ceRNAs) to regulate gene expression by microRNAs (miRNAs) sponge effects** (Figure 2A). miRNAs are important post-transcriptional regulators of gene expression that act by direct base pairing to target sites within untranslated regions of messenger RNAs (mRNAs) (52, 53). circRNAs contain miRNA response elements (MREs) that promote the binding between circRNAs and miRNAs. This binding can decrease the level of functional miRNAs and increase the expression of miRNA targets (53, 54). It has been reported that circRNAs regulate cell function by acting as miRNA sponges. For example, circFOXK2 promotes cell growth, migration, invasion, and apoptosis by binding to multiple sites and functioning as a sponge for miR-942 (55). Similarly, circRNA_100876 regulates the progression of triple-negative breast cancer by functioning as a sponge for miR-136 (56). Other circRNAs, such as circSLC26A4, circRNA_0000253, and circRNA_ANKIB1 can also function as the sponge of miR-1287-5p (57), miRNA-141-5p (58), and miR-195a-5p (59), respectively. circALMS1_6 may participate in the regulation of cardiac remodeling by functioning as a sponge for miR-133 (60).

**2) circRNAs can function as RBP sponges and RBPs can also participate in back-splicing** (Figure 2B) (61–66). RBPs are a group of proteins involved in gene transcription and translation. circRNAs can interact with RBP and inhibit their activities (66–68). circMbl absorbs MBL proteins and regulates the subsequent physiological processes (32). circPABPN1 can bind to HuR to suppress the translation of PABPN1 mRNA (69). circANRIL competitively recruits PES1 to inhibit ribosome biogenesis (70). circFoxo3 interacts with different RBPs to participate in the processes of cardiomyocyte senescence and cell cycle progression (71). circAmotl1 can protect cardiomyocytes and promote cell proliferation and wound healing by binding to PDK1, AKT1, and STAT3 (72, 73). The studies confirm the involvement of circRNAs in post-transcriptional regulation by chelating RBP. In 2020, Okholm et al. (74) conducted an extensive screen of circRNA-RBP interactions and analyzed circRNA-RBP interactions using a large set of eCLIP data with binding sites of 150 RBPs in the ENCODE cell lines HepG2 and K562 with deep-sequenced total RNA samples. Through this study, they confirmed the interactions between circCDYL and RBPs in bladder cancer cells.

**3) circRNAs mediated regulation of the transcription of parental genes** (Figure 2C). For example, circβ-catenin can produce a novel 370-amino acid β-catenin isoform using the start codon as the linear β-catenin mRNA transcript and terminates translation at a new stop codon created by circularization (75). ci-ankrd52 is a circular intronic RNA that is abundant in the nucleus and has little enrichment at microRNA target sites. ci-ankrd52 can bind to the transcription sites and acts as a positive regulator of Pol II transcription (28). For the circRNAs that interact with RNA polymerase II, exons are typically circularized with introns which are ‘retained' between exons. These circRNAs are termed exon-intron circRNAs or EIciRNAs. They are mainly localized in the nucleus and interact with U1 snRNP to promote transcription of their parental genes (29).

**4) circRNAs can be translated into proteins via some modification** (Figure 2D)**. As we know, the translation is performed by ribosomes and involves initiation, elongation, termination and ribosome recycling**. Base-modification N6-methyladenosine (m6A) is a common form of base modification in RNAs. It can promote efficient initiation of protein translation from circRNAs in human cells. Legnini et al. revealed that m6A-driven translation of circRNAs is widespread with hundreds of endogenous circRNAs carrying the translation potential (76). circ-ZNF609 is an example of a protein-coding circRNA in eukaryotes. It is related to heavy polysomes and can be translated into a protein in a splicing-dependent and cap-independent manner (77). circRNAs play biological functions through the formation of complexes with proteins; otherwise, a novel protein circFAM188B-103aa encoded by circFAM188B that promotes the proliferation but inhibits the differentiation of chicken SMSCs was identified (78). Moreover, artificial (79) and endogenous circRNAs containing an internal ribosome entry site (IRES) that directly recruits ribosomes (80) can also be translated into protein. Additionally, circRNA with an infinite ORF has hundred-fold higher productivity than linear transcript by rolling circle amplification in an IRES-independent manner (81).

**5) circRNAs may bind, store, sort, and sequester proteins to particular subcellular locations, can and act as dynamic scaffolding molecules that modulate protein-protein interactions** (Figure 2E). circRNAs can bind to RNAs and can also bind, store, sort or sequester selected proteins such as RBPs to modulate their activity or localization (82). RNA-binding protein 3 (RBM3) dynamically adjusts the proliferation of hepatocellular carcinoma cells by regulating the production of SCD-circRNA2 encoded by the 3′-UTR of the stearoyl-CoA desaturase (SCD) gene (83). Recent studies have shown that RBP quaking could also modify the formation of circRNA through forming RNA-protein complexes (RPCs) (36). In addition to interacting with RBPs, circRNAs can function as protein sponges by adsorbing one or more proteins in binding sites, thereby acting as protein scaffolding by the mediating interaction between proteins. For example, CircFOXO3 could mediate the formation of circFOXO3-p21-CDK2 ternary complex and then serve as scaffolding, affecting the cell cycle progression of cancer (84).

## Sequencing of circRNA

RNA-seq emerges as a powerful research tool to study the expression and function of non-coding RNAs including circRNAs (85). The technology of circRNA-seq generally includes library construction, computer sequencing, data analysis and processing, and function prediction (86). Either full transcriptome or circRNA profiling may be used to sequence circRNAs. The full transcriptome profiling is aimed to explore the expression patterns of both coding and non-coding RNA. This approach is suitable for the study of the biological function of circRNA. The circRNA profiling is focused on enriched circRNAs and this approach is appropriate to discover unknown circRNAs. Technically, the main difference between the two approaches is the construction of the sequencing library. The circRNA sequencing library not only requires the removal of most rRNA and poly (A), but also requires the use of ribonuclease RNase R to remove the interference of linear RNA. It has been reported that the abundance of circRNAs decreases after de-linear RNA because some circRNAs are sensitive to RNase R mediated digestion (87, 88). It is worthy to note that the alternative splicing of circRNA requires distinguishing the source of sense and antisense chains in the sequencing results. Therefore, constructing a chain-specific library is ideal as it may improve the accuracy of circRNA sequencing (87). Thus far, more than 100,000 unique human circRNAs have been discovered (89, 90).

After obtaining the circRNA sequencing data, the prediction and identification of circRNAs were carried out based on the identification software such as find circ, CIRCexplorer2, and CIRI (91–93). Real-time fluorescence quantitative PCR (quantitative real-time PCR, qRT-PCR), Northern blot hybridization (Northern blot), *in situ* hybridization (*In situ* hybridization, ISH), RPAD (RNase R treatment, polyadenylation, and poly (A) + RNA Depletion) and other techniques are used to validate the data of circRNA sequencing (94–97).

Microarray chip is another efficient tool for circRNA analysis, and it is commonly used in clinics for disease diagnosis. Compared to RNA-seq, microarray chip analysis is different in the following aspects: (2) microarray analysis of circRNA requires a known reference sequence, while RNA-seq can be utilized to analyze unknown circRNAs; (3) microarray chip analysis can be used to quantify circRNA expression when comparing with RNA-seq (98); and (4) microarray chip analysis can efficiently detect reverse splice site sequences and obtain a larger number of circRNAs than RNA-seq (99). However, some limitations of microarray chip analysis include: (2) high total RNA input is required during sample pretreatment; and (3) unlike full transcriptome sequencing, microarray chip does not give the linear RNA data (100). If the reference sequence is unknown, many studies usually use RNA-seq to determine the full transcriptional sequence, then analyze the circRNAs by microarray.

## circRNA and CVDs

With the development of deep sequencing technology, we can now understand the types and differential expression of circRNAs and their associated miRNAs in cardiovascular tissues (101–103). Many circRNAs have been reported to be associated with CVDs and their expression pattern are different between healthy and diseased human hearts (15, 19, 104, 105). Quantitative proteomics may be used to discover the regulatory networks of circRNAs in cardiovascular tissues (106). Here, we summarized recent publications on the roles of circRNAs in the development and treatment of CVDs (Table 1).

**Table 1:** Circular RNAs in cardiovascular disease and regeneration.

**CVD type**	**CircRNAs**	**Source**	**Action mechanism**	**Regulation**	**References**
Cardiac Hypertrophy	circRNA wwp1	Mouse myocardial tissue	Sponge miR-23a	Down	(107).
	circRNA HRCR	Mouse heart tissue	Sponge miR-223	Down	(108).
Myocardial Fibrosis	circ_LAS1L	Human cardiac fibroblasts	Sponge miR-125b	Down	(12)
	circ_BMP2K	Cardiac fibroblast	Sponge miR-455-3p	Down	(109)
	circPAN3	Rat myocardial tissue	Sponge miR-221	Up	(110)
	circ ACAP2	Rat cardiomyocytes cell lines	Sponge miR-29	Up	(109).
	circRNA_010567	Mouse cardiac fibroblasts	Sponge miR-141	Up	(111)
	circRNA_000203	Mouse cardiac fibroblasts	Sponge miR-26b-5p	Up	(112)
	circNFIB	Mouse heart tissue	Sponge miR-433	Down	(113)
	circHIPK3	Mouse cardiac fibroblasts	Sponge miR-29b-3p	UP	(114)
Cardiomyocyte	circ-ITCH	Rat cardiomyocytes cell lines	Sponge miR-17-5p	Down	(115)
Apoptosis	circSAMD4A	Rat cardiomyocytes cell lines	Sponge miR-138-5p	Up	(116)
	circPAN3	Rat myocardial tissue / Rat	Sponge miR-31-5p	Down	(117)
		cardiomyocytes cell lines			
		Rat myocardial tissue / Rat	Sponge miR-421	Down	(118)
		Mouse myocardial tissue			
	circDLGAP4	Human endothelial cell lines	Sponge miR-143	Down	(119)
	circFndc3b	Mouse myocardial tissue	Sponge RBP FUS	Up	(8)
	circMAT2B	Rat cardiomyocytes cell lines	Sponge miR-134	Up	(120)
	circNFIX	Rat cardiomyocytes cell lines	Unknown	Down	(121)
	circ ACAP2	Rat cardiomyocytes cell lines	Sponge miR-29	Up	(109)
	hsa_circ_0000064	Rat myocardial tissue	Unknown	Up	(122)
Coronary Heart	circDHCR24	Human aortic vascular	Sponge miR-149	Up	(20)
Disease		smooth muscle cell			
	circZNF609	Human peripheral blood	Unknow	Down	(19)
	circMAP3K5	Human coronary artery	Sponge miR-22-3p	Down	(23)
		smooth muscle cells			
	circRNA-100338	Human endothelial cell lines	Sponge miRNA-200a-3p	Down	(17)
	hsa_circ_0089378	Plasma	Sponge hsa-miR-130a-3p	Up	(21)
	hsa_circ_0083357				
	hsa_circ_0082824				
	hsa_circ_0068942				
	hsa_circ_0057576				
	hsa_circ_0054537				
	hsa_circ_0051172				
	hsa_circ_0032970				
	hsa_circ_0006323				
Heart Failure	hsa_circ_0005565	Human heart tissue	Unknown	Up	(16)
	hsa_circ_0097435	Human peripheral blood	Sponge Hsa_miR_609	Up	(15)
			Sponge Hsa_miR_1294		
			Sponge Hsa_miR_6799_5P		
			Sponge Hsa_miR_5000_5P		
			Sponge Hsa_miR_96_5P		
Myocardial	circTLK1	Mouse myocardial tissue	Sponge miR-214	Up	(11)
Regeneration	circRNA CDR1as	Pig myocardial tissue	Sponge miR-7	Up	(10)
	circ003593	Cardiomyocytes cell lines	Unknown	Up	(13)
	circ-0001273	Human umbilical cord mesenchymal	Unknown	Down	(9)
		stem cells (UMSCs)			
	circCDYL	Mouse myocardial tissue	Sponge miR-4793-5p	Down	(14)
	circFASTKD1	Human endothelial cell lines	Sponge miR-106a	Down	(123)
	hsa_circ_0007623	Human endothelial cell lines	Sponge miR-297	Up	(124)
	circHipk3	Mouse heart tissue	Sponge miR-133a	Up	(104)
	circRNA_0001379	Mouse myocardial tissue	Sponge miR-17-5p	Up	(103)

**1) Cardiac hypertrophy**. Cardiac hypertrophy is the heart's response to a variety of extrinsic and intrinsic stimuli that impose increased biomechanical stress and can be caused by various cardiovascular diseases. circRNA wwp1 exerts inhibitory roles of cardiac hypertrophy via down-regulation of ANF and miR-23a in isoproterenol hydrochloride-induced cardiac hypertrophy (107). A circRNA HRCR functions as an endogenous miR-223 sponge to sequester and inhibit miR-223 activity, resulting in an increase of ARC expression and protection of the heart from pathological hypertrophy and heart failure (108). Modulation of circRNAs levels may provide a promising therapeutic target for the treatment of cardiac hypertrophy.

**2) Cardiac fibrosis**. Activation and phenotypical transition of cardiac fibroblasts contribute to cardiac fibrosis. It was reported that circ_BMP2K enhances the regulatory effects of miR-455-3p on its target gene SUMO1 which leads to the inhibition of TGF-β1 or Ang II to induce the activation of cardiac fibroblasts (109). circRNA_010567, circRNA_000203, and circHIPK3 were upregulated in Angiotensin-II (Ang-II)-induced activation of cardiac fibroblasts (111, 112, 114). circNFIB was downregulated in TGF-β induced activation of primary adult cardiac fibroblasts (113). Some studies showed that targeting circRNAs improve myocardial ischemic and reperfusion injuries by attenuating myocardial fibrosis. For example, circ_LAS1L is down-regulated in patients with acute myocardial infarction and regulates cardiac fibroblast activation, growth, and migration by inhibiting miR-125b/SFRP5 pathway (12). circPAN3 knockdown attenuated autophagy-mediated cardiac fibrosis after myocardial infarction via miR-221/FoxO3/ATG7 axis (110). The roles of circRNAs in cardiac fibrosis has been summarized in a recent review article (54).

**3) Cardiomyocyte apoptosis**. It was reported that some circRNAs may be involved in injury-induced cardiomyocyte apoptosis. For example, circRNA ITCH mediates H_2_O_2_-induced myocardial cell apoptosis by upregulating miR-17-5p via wnt/β-catenin signaling pathway (115). circSAMD4A aggravates hypoxia/reoxygenation (H/R)-induced cardiomyocyte apoptosis and inflammatory response by sponging miR-138-5p (116). MicroRNA-31-5p acts as a negative regulator of circPAN3 by directly suppressing QKI in doxorubicin-induced apoptosis of cardiomyocytes (117). circPAN3 also ameliorates myocardial ischemia and reperfusion injury by regulating miR-421/Pink1 axis-mediated suppression of autophagy (118). HECTD1 overexpression increases cell viability and decreases cell apoptosis and migration, and circDLPAG4/HECTD1 mediates ischemia/reperfusion injury in endothelial cells via ER stress (119). Down-regulation of circFndc3b was observed in mice with myocardial infarction, and overexpression of circFndc3b increases angiogenic activity and reduces cell apoptosis in cardiac endothelial cells and cardiomyocytes which led to improved left ventricular functions (8). Other studies showed that miR-133 was regulated by circMAT2B. CircMAT2B knockdown attenuates oxygen-glucose deprivation-induced injury through up-regulating miR-133 in H9c2 cells (120). circNFIX can serve as a pro-apoptosis factor in cardiomyocytes (121). The expression of circ ACAP2 is induced by myocardial infarction which leads to increased cardiomyocyte apoptosis by sponging miR-29 (109). Salidroside inhibits apoptosis and autophagy of cardiomyocytes by regulation of circular RNA hsa_circ_0000064 in cardiac ischemia-reperfusion injury (122). These data suggest that circRNAs may be the new targets for designing cardioprotective treatments against cardiomyocyte death.

**4) Coronary heart disease**. circRNAs are involved in the pathogenesis of atherosclerosis and coronary heart disease. It has been reported that suppression of circDHCR24 alleviates aortic smooth muscle cell proliferation and migration by targeting miR-149-5p/MMP9 axis in human aortic vascular smooth muscle cells after PDGF-BB treatment (20). Recent studies showed that the expression level of circZNF609 in peripheral blood leukocytes of patients with coronary artery disease was significantly decreased, and circZNF609 regulates the release of inflammatory cytokines such as IL-6, IL-10, and TNF-α by serving as sponges to different miRNAs that control the expression of these cytokines (19). circMAP3K5 was downregulated in patients with coronary heart disease and acted as a microRNA-22-3p sponge to promote resolution of intimal hyperplasia via TET2-mediated smooth muscle cell differentiation (23). circRNA-100338 may induce angiogenesis after myocardial ischemia-reperfusion injury by sponging miR-200a-3p in human coronary endothelial cells (17). The level of hsa_circ_0001445 in plasma was associated with the severity of coronary atherosclerosis. *In vitro*, hsa_circ_0001445 was downregulated in extracellular vesicles secreted by human coronary smooth muscle cells upon exposure to atherogenic conditions (22). The 3 circRNAs (hsa_circ_0016868, hsa_circ_0001364, hsa_circ_0006731) have been verified by the coronary artery segments Sanger sequencing obtained from an 81-year-old male patient with the sudden death of myocardial infarction (18). In addition, a recent study on the differential expression of circRNAs in plasma samples from patients with coronary heart disease identified 9 circRNAs that promote the expression of transient receptor potential cation channel subfamily M member 3 by inhibiting hsa-miR-130a-3p (21). These data demonstrate that circRNAs play important roles in the pathogenesis of coronary heart disease and may serve as diagnostic or therapeutic targets for coronary heart disease.

**5) Heart failure**. Despite the detailed roles of circRNAs in the progression of human heart failure remains elusive, recent high-throughput sequencing studies identified many circRNAs with changed expression in patients with heart failure. The top highly expressed EAT circRNAs corresponded to genes involved in cell proliferation and inflammatory responses. A recent study on circRNA expression profile in epicardial adipose tissue in patients with heart failure showed that EAT circRNAs may contribute to the pathogenesis of metabolic disorders (16). Another study showed that the upregulation of Hsa_circ_0097435 contributes to the pathogenesis of heart failure via sponging multiple microRNAs (15).

**6) Cardiac regeneration and repair**. Recent studies indicated that modulating circRNA activity may have great therapeutic potential for myocardial regeneration and repair. Up- or down-regulation of circRNAs and miRNAs and circRNA-miRNA coexpression had been shown to change the expression of the genes associated with myocardial ischemia and reperfusion injuries (101, 103). It has been reported that circRNAs can regulate inflammatory factors to improve myocardial ischemia and reperfusion injury. The circTLK1 exacerbates myocardial ischemia and reperfusion injury via targeting miR-214/RIPK1 through TNF signaling pathway (11). circ003593 has also been shown to confer cardioprotection through NLRP3 inflammasome myocardial infarct rats (13). circRNA CDR1as was identified in pig hearts. Elevated circRNA CDR1as in the infarction region of the pig heart is negatively associated with infarct size and positively associated with improved heart function (10). circ-0001273 can remarkably inhibit myocardial cell apoptosis and promote repair in myocardial infarction hearts (9). circCDYL was downregulated in myocardial tissues and hypoxia myocardial cells after acute myocardial infarction. circCDYL overexpression and downregulation can promote and inhibit the proliferation of cardiomyocytes *in vitro*, respectively. Additionally, circCDYL can promote the proliferation of cardiomyocytes through the miR-4793-5p/APP pathway (14). The downregulation of circFASTKD1 induces angiogenesis and improves cardiac function and repair after myocardial infarction (123). Hsa_circ_0007623 can bind to miR-297 and acts as a sponge of microRNA-297 which promotes cardiac repair after acute myocardial ischemia and protects cardiac function (124). circHipk3 was overexpressed in the fetal or neonatal heart of mice and functioned to promote the proliferation of cardiomyocyte and endothelial cells which leads to angiogenesis. Further study showed that circHipk3 regulates cardiac regeneration in mice post myocardial infarction by interacting with Notch1 and miR-133a (104). These findings highlight the physiological role of circRNAs in cardiac repair and indicate that modulation of circRNA may represent a potential strategy to promote cardiac function and remodeling after myocardial injuries.

## Future Perspectives

The studies we discussed in the paper highlight the significance of circRNAs in the pathogenesis of CVDs. circRNAs are stable and abundantly present in the circulatory system which enables them to serve as biomarkers for the diagnosis and treatment of CVDs; however, there are some critical issues to be addressed. Firstly, there is no reliable methodology for the detection of circRNAs. In terms of circRNA detection, newer, simpler, and more reliable methods will be expected to appear. This will provide convenience for us to study circRNAs, facilitate the faster output of research results, and obtain more target circRNAs with diagnostic and therapeutic significance. Secondly, some circRNA biomarkers come from small samples and populations. This makes us question the reliability and representativeness of the research results. Thirdly, the mechanism underlying circRNA functions in the cardiovascular system remain largely elusive. For the function and mechanism studies, the current research methods are limited and difficult to operate. New research protocols need to be further explored. Rapid development can be achieved only by breaking through the technological bottleneck in the field of circRNA research. Lastly, there is a lack of efficient approaches for modulating circRNA expression in the cardiovascular system. It is supposed to be that in the future there will be new and more diverse methods in modulating the overexpression and inhibition of circRNAs. This will promote the development of the mechanism underlying circRNA functions. Then the research results can be quickly used for clinical diagnosis and treatment in the field of vascular diseases.

## Author Contributions

All authors listed have made a substantial, direct and intellectual contribution to the work, and approved it for publication.

## Conflict of Interest

The authors declare that the research was conducted in the absence of any commercial or financial relationships that could be construed as a potential conflict of interest.

## References

[1] Sanger HL Riesner D and Gross HJ Kleinschmidt AK. Viroids are single-stranded covalently closed circular RNA molecules existing as highly base-paired rod-like structures. Proc Natl Acad Sci USA. (1976) 73:3852–6. 10.1073/pnas.73.11.3852 1069269 PMC431239

[2] Hsu MT Coca-Prados M. Electron microscopic evidence for the circular form of RNA in the cytoplasm of eukaryotic cells. Nature. (1979) 280:339–40. 10.1038/280339a0 460409

[3] Capel B Nicolis AS Hacker S Walter M Koopman P Goodfellow P. Lovell-Badge R. Cell-Circular transcripts of the testis-determining gene Sry in adult mouse testis. Cell. (1993) 73:1019–30. 10.1016/0092-8674(93)90279-Y 7684656

[4] Salzman J Gawad C Wang PL Lacayo N Brown PO. Circular RNAs are the predominant transcript isoform from hundreds of human genes in diverse cell types. PLoS One. (2012) 7:e30733. 10.1371/journal.pone.0030733 22319583 PMC3270023

[5] Hansen TB Veno MT Damgaard CK Kjems J. Comparison of circular RNA prediction tools. Nucleic Acids Res. (2016) 44:e58. 10.1093/nar/gkv1458 26657634 PMC4824091

[6] Li S Li X Xue W Zhang L Yang LZ Cao SM. Screening for functional circular RNAs using the CRISPR-Cas13 system. Nat Methods. (2021) 18:51–59. 10.1038/s41592-020-01011-4 33288960

[7] Jakobi T Czaja-Hasse LF Reinhardt R Dieterich C. Profiling and validation of the circular RNA repertoire in adult murine hearts. Gen Prot Bioinform. (2016) 14:216–23. 10.1016/j.gpb.2016.02.003 27132142 PMC4996846

[8] Garikipati VNS Verma SK Cheng Z Liang D Truongcao MM Cimini M. Circular RNA CircFndc3b modulates cardiac repair after myocardial infarction via FUS/VEGF-A axis. Nat Commun. (2019) 10:4317. 10.1038/s41467-019-11777-7 31541092 PMC6754461

[9] Lei D Wang Y Zhang L Wang Z. Circ_0010729 regulates hypoxia-induced cardiomyocyte injuries by activating TRAF5 via sponging miR-27a-3p. Life Sci. (2020) 262:118511. 10.1016/j.lfs.2020.118511 33010282

[10] Mester-Tonczar J Winkler J Einzinger P Hasimbegovic E Kastner N Lukovic D. Association between circular RNA CDR1as and post-infarction cardiac function in pig ischemic heart failure: influence of the anti-fibrotic natural compounds bufalin and lycorine. Biomolecules. (2020) 10:1180. 10.3390/biom10081180 32823854 PMC7463784

[11] Song YF Zhao L Wang BC Sun JJ Hu JL Zhu XL. The circular RNA TLK1 exacerbates myocardial ischemia/reperfusion injury via targeting miR-214/RIPK1 through TNF signaling pathway. Free Radic Biol Med. (2020) 155:69–80. 10.1016/j.freeradbiomed.2020.05.013 32445866

[12] Sun LY Zhao JC Ge XM Zhang H Wang CM Bie ZD. Circ_LAS1L regulates cardiac fibroblast activation, growth, and migration through miR-125b/SFRP5 pathway. Cell Biochem Funct. (2020) 38:443–50. 10.1002/cbf.3486 31950540

[13] Xiao Y Oumarou DB Wang S Liu Y. Circular RNA involved in the protective effect of *Malva sylvestris* L. on myocardial ischemic/re-perfused *Injury Front Pharmacol*. (2020) 11:520486. 10.3389/fphar.2020.520486 33101012 PMC7546788

[14] Zhang M Wang Z Cheng Q Wang Z Lv X Wang Z. Circular RNA (circRNA) CDYL induces myocardial regeneration by ceRNA amyocardial infarction. Med Sci Monit. (2020) 26:e923188. 10.12659/MSM.923188 32522972 PMC7304314

[15] Han J Zhang L Hu L Yu H Xu F Yang B. Circular RNA-expression profiling reveals a potential role of Hsa_circ_0097435 in heart failure via sponging multiple MicroRNAs. Front Genet. (2020) 11:212. 10.3389/fgene.2020.00212 32211036 PMC7076158

[16] Zheng ML Du XP Zhao L Yang XC. Expression profile of circular RNAs in epicardial adipose tissue in heart failure. Chin Med J. (2020) 133:2565–72. 10.1097/CM9.0000000000001056 32852391 PMC7722589

[17] Chang H Wu ZB Zhang L. Circ-100338 induces angiogenesis after myocardial ischemia-reperfusion injury by sponging miR-200a-3p. Eur Rev Med Pharmacol Sci. (2020) 24:6323–32. 10.26355/eurrev_202006_21530 32572929

[18] Hou C Gu L Guo Y Zhou Y Hua L Chen J. Association between circular RNA expression content and severity of coronary atherosclerosis in human coronary artery. J Clin Lab Anal. (2020) 34:e23552. 10.1002/jcla.23552 32889742 PMC7755800

[19] Liang B Li M Deng Q Wang C Rong J He S. CircRNA ZNF609 in peripheral blood leukocytes acts as a protective factor and a potential biomarker for coronary artery disease. Ann Transl Med. (2020) 8:741. 10.21037/atm-19-4728 32647666 PMC7333115

[20] Peng W Li T Pi S Huang L Liu Y. Suppression of circular RNA circDHCR24 alleviates aortic smooth muscle cell proliferation and migration by targeting miR-149-5p/MMP9 axis. Biochem Biophys Res Commun. (2020) 529:753–9. 10.1016/j.bbrc.2020.06.067 32736703

[21] Pan RY Liu P Zhou HT Sun WX Song J Shu J. Circular RNAs promote TRPM3 expression by inhibiting hsa-miR-130a-3p in coronary artery disease patients. Oncotarget. (2020) 8:60280–90. 10.18632/oncotarget.19941 28947970 PMC5601138

[22] Vilades D Martinez-Camblor P Ferrero-Gregori A Bar C Lu D Xiao K. Plasma circular RNA hsa_circ_0001445 and coronary artery disease: performance as a biomarker. FASEB J. (2020) 34:4403–14. 10.1096/fj.201902507R 31999007

[23] Zeng Z Xia L Fan S Zheng J Qin J Fan X. Circular RNA CircMAP3K5 acts as a MicroRNA-22-3p sponge to promote resolution of intimal hyperplasia via TET2-mediated smooth muscle cell differentiation. Circulation. (2021) 143:354–71. 10.1161/CIRCULATIONAHA.120.049715 33207953

[24] Vausort M Salgado-Somoza A Zhang L Leszek P Scholz M Teren A. Myocardial infarction-associated circular RNA predicting left ventricular dysfunction. J Am Coll Cardiol. (2016) 68:1247–8. 10.1016/j.jacc.2016.06.040 27609688

[25] Zhao Z Li X Gao C Jian D Hao P Rao L. Peripheral blood circular RNA hsa_circ_0124644 can be used as a diagnostic biomarker of coronary artery disease. Sci Rep. (2017) 7:39918. 10.1038/srep39918 28045102 PMC5206672

[26] Zhang J Xu Y Xu S Liu Y Yu L Li Z. Plasma circular RNAs, Hsa_circRNA_025016, predict postoperative atrial fibrillation after isolated off-pump coronary artery bypass grafting. J Am Heart Assoc. (2018) 7:6642. 10.1161/JAHA.117.006642

[27] Jeck WR Sorrentino JA Wang K Slevin MK Burd CE Liu J. Circular RNAs are abundant, conserved, and associated with ALU repeats. RNA. (2013) 19:141–157. 10.1261/rna.035667.112 23249747 PMC3543092

[28] Zhang Y Zhang XO Chen T Xiang JF Yin QF Xing YH. Circular intronic long noncoding RNAs. Mol Cell. (2013) 51:792–806. 10.1016/j.molcel.2013.08.017 24035497

[29] Li Z Huang C Bao C Chen L Lin M Wang X. Exon-intron circular RNAs regulate transcription in the nucleus. Nat Struct Mol Biol. (2015) 22:256–64. 10.1038/nsmb.2959 25664725

[30] Schmidt CA Matera AG. tRNA introns: presence, processing, and purpose. Wiley Interdiscip Rev RNA. (2020) 11:e1583. 10.1002/wrna.1583 31883233

[31] Zhang XO Wang HB Zhang Y Lu X Chen LL Yang L. Complementary sequence-mediated exon circularization. Cell. (2014) 159:134–47. 10.1016/j.cell.2014.09.001 25242744

[32] Ashwal-Fluss R Meyer M Pamudurti NR Ivanov A Bartok O Hanan M. circRNA biogenesis competes with pre-mRNA splicing. Mol Cell. (2014) 56:55–66. 10.1016/j.molcel.2014.08.019 25242144

[33] Petkovic S Muller S. RNA circularization strategies *in vivo* and *in vitro*. Nucleic Acids Res. (2015) 43:2454–65. 10.1093/nar/gkv045 25662225 PMC4344496

[34] Shi Y He R Yang Y He Y Shao K Zhan L. Circular RNAs: Novel biomarkers for cervical, ovarian and endometrial cancer. Oncol Rep. (2020) 44:1787–98. 10.3892/or.2020.7780 33000238 PMC7551080

[35] Bachmayr-Heyda A Reiner AT Auer K Sukhbaatar N Aust S Bachleitner-Hofmann T. Correlation of circular RNA abundance with proliferation–exemplified with colorectal and ovarian cancer, idiopathic lung fibrosis, and normal human tissues. Sci Rep. (2015) 5:8057. 10.1038/srep08057 25624062 PMC4306919

[36] Conn SJ Pillman KA Toubia J Conn VM Salmanidis M Phillips CA. The RNA binding protein quaking regulates formation of circRNAs. Cell. (2015) 160:1125–1134. 10.1016/j.cell.2015.02.014 25768908

[37] Kramer MC Liang D Tatomer DC Gold B March ZM Cherry S. Combinatorial control of Drosophila circular RNA expression by intronic repeats, hnRNPs, SR proteins. Genes Dev. (2015) 29:2168–82. 10.1101/gad.270421.115 26450910 PMC4617980

[38] Li X Liu CX Xue W Zhang Y Jiang S Yin QF. Coordinated circRNA biogenesis and function with NF90/NF110 in viral infection. Mol Cell. (2017) 67:214–27 e217. 10.1016/j.molcel.2017.05.023 28625552

[39] Salzman J Chen RE Olsen MN Wang PL Brown PO. Cell-type specific features of circular RNA expression. PLoS Genet. (2013) 9:e1003777. 10.1371/annotation/f782282b-eefa-4c8d-985c-b1484e845855 24039610 PMC3764148

[40] Xu T Wu J Han P Zhao Z Song X. Circular RNA expression profiles and features in human tissues: a study using RNA-seq data. BMC Genomics. (2017) 18:680. 10.1186/s12864-017-4029-3 28984197 PMC5629547

[41] Zeng X Lin W Guo M Zou Q. A comprehensive overview and evaluation of circular RNA detection tools. PLoS Comput Biol. (2017) 13:e1005420. 10.1371/journal.pcbi.1005420 28594838 PMC5466358

[42] Li XF Lytton J. A circularized sodium-calcium exchanger exon 2 transcript. J Biol Chem. (1999) 274:8153–60. 10.1074/jbc.274.12.8153 10075718

[43] Memczak S Jens M Elefsinioti A Torti F Krueger J Rybak A. Circular RNAs are a large class of animal RNAs with regulatory potency. Nature. (2013) 495:333–8. 10.1038/nature11928 23446348

[44] Li Y Zhang J Huo C Ding N Li J Xiao J. Dynamic Organization of lncRNA and circular RNA regulators collectively controlled cardiac differentiation in humans. EBioMedicine. (2017) 24:137–46. 10.1016/j.ebiom.2017.09.015 29037607 PMC5652025

[45] Siede D Rapti K Gorska AA Katus HA Altmuller J Boeckel JN. Identification of circular RNAs with host gene-independent expression in human model systems for cardiac differentiation and disease. J Mol Cell Cardiol. (2017) 109:48–56. 10.1016/j.yjmcc.2017.06.015 28676412

[46] Guo JU Agarwal V Guo H Bartel DP. Expanded identification and characterization of mammalian circular RNAs. Genome Biol. (2014) 15:409. 10.1186/s13059-014-0409-z 25070500 PMC4165365

[47] Werfel S Nothjunge S Schwarzmayr T Strom TM Meitinger T Engelhardt S. Characterization of circular RNAs in human, mouse and rat hearts. J Mol Cell Cardiol. (2016) 98:103–7. 10.1016/j.yjmcc.2016.07.007 27476877

[48] Aufiero S Reckman YJ Pinto YM Creemers EE. Circular RNAs open a new chapter in cardiovascular biology. Nat Rev Cardiol. (2019) 16:503–14. 10.1038/s41569-019-0185-2 30952956

[49] Lim TB Lavenniah A Foo RS. Circles in the heart and cardiovascular system. Cardiovasc Res. (2020) 116:269–78. 10.1093/cvr/cvz227 31552406

[50] Gupta SK Garg A Bar C Chatterjee S Foinquinos A Milting H. Quaking inhibits doxorubicin-mediated cardiotoxicity through regulation of cardiac circular RNA expression. Circ Res. (2018) 122:246–54. 10.1161/C.I.R.C.R.E.S.A.H.A.117.311335 29133306 PMC5771684

[51] Dong R Zhang XO Zhang Y Ma XK Chen LL Yang L. CircRNA-derived pseudogenes. Cell Res. (2016) 26:747–50. 10.1038/cr.2016.42 27021280 PMC4897178

[52] Bartel DP. MicroRNAs: target recognition and regulatory functions. Cell. (2009) 136:215–33. 10.1016/j.cell.2009.01.002 19167326 PMC3794896

[53] Hansen TB Jensen TI Clausen BH Bramsen JB Finsen B Damgaard CK. Natural RNA circles function as efficient microRNA sponges. Nature. (2013) 495:384–8. 10.1038/nature11993 23446346

[54] Zhang L Zhang Y Wang Y Zhao Y Ding H Li P. Circular RNAs: functions and clinical significance in cardiovascular disease. Front Cell Dev Biol. (2020) 8:584051. 10.3389/fcell.2020.584051 33134301 PMC7550538

[55] Wong CH Lou UK Li Y Chan SL Tong JH To KF. CircFOXK2 promotes growth and metastasis of pancreatic ductal adenocarcinoma by complexing with RNA-binding proteins and sponging MiR-942. Cancer Res. (2020) 80:2138–49. 10.1158/0008-5472.CAN-19-3268 32217695

[56] Yu X Xiao W Song H Jin Y Xu J Liu X. CircRNA_100876 sponges miR-136 to promote proliferation and metastasis of gastric cancer by upregulating MIEN1 expression. Gene. (2020) 748:144678. 10.1016/j.gene.2020.144678 32305633

[57] Ji F Du R Chen T Zhang M Zhu Y Luo X. Circular RNA circSLC26A4 accelerates cervical cancer progression via miR-1287-5p/HOXA7 axis. Mol Ther Nucleic Acids. (2020) 19:413–20. 10.1016/j.omtn.2019.11.032 31896069 PMC6940609

[58] Song J Chen ZH Zheng CJ Song KH Xu GY Xu S. Exosome-Transported circRNA_0000253 competitively adsorbs MicroRNA-141-5p and increases IDD. Mol Ther Nucleic Acids. (2020) 21:1087–99. 10.1016/j.omtn.2020.07.039 32858458 PMC7473879

[59] Li C Mu J Shi Y Xin H. LncRNA CCDC26 interacts with CELF2 protein to enhance myeloid leukemia cell proliferation and invasion via the circRNA_ANKIB1/miR-195-5p/PRR11 axis. Cell Transplant. (2021) 30: 80. 10.1177/0963689720986080 33439746 PMC7809300

[60] Dong K He X Su H Fulton DJR Zhou J. Genomic analysis of circular RNAs in heart. BMC Med Genomics. (2020) 13:167. 10.1186/s12920-020-00817-7 33160353 PMC7648966

[61] Pan X Shen HB. RNA-protein binding motifs mining with a new hybrid deep learning based cross-domain knowledge integration approach. BMC Bioinform. (2017) 18:136. 10.1186/s12859-017-1561-8 28245811 PMC5331642

[62] Pan X Rijnbeek P Yan J Shen HB. Prediction of RNA-protein sequence and structure binding preferences using deep convolutional and recurrent neural networks. BMC Gen. (2018) 19:511. 10.1186/s12864-018-4889-1 29970003 PMC6029131

[63] Pan X Shen HB. Predicting RNA-protein binding sites and motifs through combining local and global deep convolutional neural networks. Bioinformatics. (2018) 34:3427–36. 10.1093/bioinformatics/bty364 29722865

[64] Wang Z Lei X Wu FX. Identifying cancer-specific circRNA-RBP binding sites based on deep learning. Molecules. (2019) 24:35. 10.3390/molecules24224035 31703384 PMC6891306

[65] Bhuyan R Bagchi A. Prediction of the differentially expressed circRNAs to decipher their roles in the onset of human colorectal cancers. Gene. (2020) 762:145035. 10.1016/j.gene.2020.145035 32777531

[66] Wawrzyniak O Zarebska Z Kuczynski K Gotz-Wieckowska A Rolle K. Protein-related circular RNAs in human pathologies. Cells. (2020) 9:1841. 10.3390/cells9081841 32781555 PMC7463956

[67] Tang Q Hann SS. Biological roles and mechanisms of circular RNA in human cancers. Onco Targets Ther. (2020) 13:2067–92. 10.2147/OTT.S233672 32210574 PMC7069569

[68] Zang J Lu D Xu A. The interaction of circRNAs and RNA binding proteins: An important part of circRNA maintenance and function. J Neurosci Res. (2020) 98:87–97. 10.1002/jnr.24356 30575990

[69] Abdelmohsen K Panda AC Munk R Grammatikakis I Dudekula DB De S. Identification of HuR target circular RNAs uncovers suppression of PABPN1 translation by CircPABPN1. RNA Biol. (2017) 14:361–9. 10.1080/15476286.2017.1279788 28080204 PMC5367248

[70] Holdt LM Stahringer A Sass K Pichler G Kulak NA Wilfert W. Circular non-coding RNA ANRIL modulates ribosomal RNA maturation and atherosclerosis in humans. Nat Commun. (2016) 7:12429. 10.1038/ncomms12429 27539542 PMC4992165

[71] Du WW Yang W Chen Y Wu ZK Foster FS Yang Z. Foxo3 circular RNA promotes cardiac senescence by modulating multiple factors associated with stress and senescence responses. Eur Heart J. (2017) 38:1402–12. 10.1093/eurheartj/ehw001 26873092

[72] Yang ZG Awan FM Du WW Zeng Y Lyu J Wu .upta S. The circular RNA interacts with STAT3, increasing its nuclear translocation and wound repair by modulating Dnmt3a and miR-17 function. Mol Ther. (2017) 25:2062–74. 10.1016/j.ymthe.2017.05.022 28676341 PMC5589065

[73] Zeng Y Du WW Wu Y Yang Z Awan FM Li X. A circular RNA binds to and activates AKT phosphorylation and nuclear localization reducing apoptosis and enhancing cardiac repair. Theranostics. (2017) 7:3842–55. 10.7150/thno.19764 29109781 PMC5667408

[74] Okholm TLH Sathe S Park SS Kamstrup AB Rasmussen AM Shankar A. Transcriptome-wide profiles of circular RNA and RNA-binding protein interactions reveal effects on circular RNA biogenesis and cancer pathway expression. Genome Med. (2020) 12:112. 10.1186/s13073-020-00812-8 33287884 PMC7722315

[75] Kalyana-Sundaram S Kumar-Sinha C Shankar S Robinson DR Wu YM Cao X. Expressed pseudogenes in the transcriptional landscape of human cancers. Cell. (2012) 149:1622–34. 10.1016/j.cell.2012.04.041 22726445 PMC3597446

[76] Yang Y Fan X Mao M Song X Wu P Zhang Y. Extensive translation of circular RNAs driven by N(6)-methyladenosine. Cell Res. (2017) 27:626–41. 10.1038/cr.2017.31 28281539 PMC5520850

[77] Legnini I Di Timoteo G Rossi F Morlando M Briganti F Sthandier O. Circ-ZNF609 is a circular RNA that can be translated and functions in myogenesis. Mol Cell. (2017) 66:22–37 e29. 10.1016/j.molcel.2017.02.017 28344082 PMC5387670

[78] Yin H Shen X Zhao J Cao X He H Han S. Circular RNA circfam188b encodes a protein that regulates proliferation and differentiation of chicken skeletal muscle satellite cells. Front Cell Dev Biol. (2020) 8:522588. 10.3389/fcell.2020.522588 33240871 PMC7677141

[79] Cy C Sarnow P. Initiation of protein synthesis by the eukaryotic translational apparatus on circular RNAs. Science. (1995) 268:415–7. 10.1126/science.7536344 7536344

[80] Macejak GD Sarnow P. Internal initiation of translation mediated by the 5′ leader of a cellular mRNA. Nature. (1991) 353:90–4. 10.1038/353090a0 1652694

[81] Abe N Hiroshima M Maruyama H Nakashima Y Nakano Y Matsuda A. Rolling circle amplification in a prokaryotic translation system using small circular RNA. Angew Chem Int Ed Engl. (2013) 52:7004–8. 10.1002/anie.201302044 23716491

[82] Hentze MW Preiss T. Circular RNAs: splicing's enigma variations. EMBO J. (2013) 32:923–5. 10.1038/emboj.2013.53 23463100 PMC3616293

[83] Dong W Dai ZH Liu FC Guo XG Ge CM Ding J. The RNA-binding protein RBM3 promotes cell proliferation in hepatocellular carcinoma by regulating circular RNA SCD-circRNA 2 production. EBioMed. (2019) 45:155–67. 10.1016/j.ebiom.2019.06.030 31235426 PMC6642271

[84] Du WW Yang W Liu E Yang Z Dhaliwal P Yang BB. Foxo3 circular RNA retards cell cycle progression via forming ternary complexes with p21 and CDK2. Nucleic Acids Res. (2016) 44:2846–58. 10.1093/nar/gkw027 26861625 PMC4824104

[85] Van Dijk EL Auger H Jaszczyszyn Y Thermes C. Ten years of next-generation sequencing technology. Trends Genet. (2014) 30:418–26. 10.1016/j.tig.2014.07.001 25108476

[86] Wang J Ren Q Hua L Chen J Zhang J Bai H. Comprehensive analysis of differentially expressed mRNA, lncRNA and circRNA and their ceRNA networks in the longissimus dorsi muscle of two different pig breeds. Int J Mol Sci. (2019) 20:1107. 10.3390/ijms20051107 30836719 PMC6429497

[87] Zhang XO Dong R Zhang Y Zhang JL Luo Z Zhang J. Diverse alternative back-splicing and alternative splicing landscape of circular RNAs. Genome Res. (2016) 26:1277–87. 10.1101/gr.202895.115 27365365 PMC5052039

[88] Dahl M Daugaard I Andersen MS Hansen TB Gronbaek K Kjems J. Enzyme-free digital counting of endogenous circular RNA molecules in B-cell malignancies. Lab Invest. (2018) 98:1657–69. 10.1038/s41374-018-0108-6 30087459 PMC6265260

[89] Glazar P Papavasileiou P Rajewsky N. circBase: a database for circular RNAs. RNA. (2014) 20:1666–70. 10.1261/rna.043687.113 25234927 PMC4201819

[90] Vo JN Cieslik M Zhang Y Shukla S Xiao L Zhang Y. The landscape of circular RNA in cancer. Cell. (2019) 176:869–81 e813. 10.1016/j.cell.2018.12.021 PMC6601354 30735636

[91] Sekar S Cuyugan L Adkins J Geiger P Liang WS. Circular RNA expression and regulatory network prediction in posterior cingulate astrocytes in elderly subjects. BMC Genomics. (2018) 19:340. 10.1186/s12864-018-4670-5 29739336 PMC5941680

[92] Sekar S Geiger P Cuyugan L Boyle A Serrano G Beach TG. Identification of circular RNAs using RNA sequencing. J Vis Exp. (2019). 10.3791/59981 31789321

[93] Zhang QL Ji XY Li HW Guo J Wang F Deng XY. Identification of circular RNAs and their altered expression under poly(I:C) challenge in key antiviral immune pathways in amphioxus. Fish Shellfish Immunol. (2019) 86:1053–7. 10.1016/j.fsi.2018.12.061 30590167

[94] Panda AC De S Grammatikakis I Munk R Yang X Piao Y. High-purity circular RNA isolation method (RPAD) reveals vast collection of intronic circRNAs. Nucleic Acids Res. (2017) 45:e116. 10.1093/nar/gkx297 28444238 PMC5499592

[95] Chuang TJ Chen YJ Chen CY Mai TL Wang YD Yeh CS. Integrative transcriptome sequencing reveals extensive alternative trans-splicing and cis-backsplicing in human cells. Nucleic Acids Res. (2018) 46:3671–91. 10.1093/nar/gky032 29385530 PMC6283421

[96] Yang Y Gao X Zhang M Yan S Sun C Xiao F. Novel role of FBXW7 circular RNA in repressing glioma tumorigenesis. J Natl Cancer Inst. (2018) 110:166. 10.1093/jnci/djx166 28903484 PMC6019044

[97] Wang L Long H Zheng Q Bo X Xiao X Li B. Circular RNA circRHOT1 promotes hepatocellular carcinoma progression by initiation of NR2F6 expression. Mol Cancer. (2019) 18:119. 10.1186/s12943-019-1046-7 31324186 PMC6639939

[98] Labaj PP Leparc GG Linggi BE Markillie LM Wiley HS Kreil DP. Characterization and improvement of RNA-Seq precision in quantitative transcript expression profiling. Bioinformatics. (2011) 27:i383–91. 10.1093/bioinformatics/btr247 21685096 PMC3117338

[99] Li S Teng S Xu J Su G Zhang Y Zhao J. Microarray is an efficient tool for circRNA profiling. Brief Bioinform. (2019) 20:1420–33. 10.1093/bib/bby006 29415187

[100] López-Jiménez E Ana R Andrés-León E. RNA sequencing and prediction tools for circular RNAs analysis. Adv Exp Med Biol. (2018) 1087:17–33. 10.1007/978-981-13-1426-1_2 30259354

[101] Lin F Yang Y Guo Q Xie M Sun S Wang X. Analysis of the molecular mechanism of acute coronary syndrome based on circRNA-miRNA network regulation. Evid Based Complement Alternat Med. (2020) 2020:1584052. 10.1155/2020/1584052 32419790 PMC7206869

[102] Liu T Zhang G Wang Y Rao M Zhang Y Guo A. Identification of Circular RNA-MicroRNA-Messenger RNA regulatory network in atrial fibrillation by integrated analysis. Biomed Res Int. (2020) 2020:8037273. 10.1155/2020/8037273 33062700 PMC7545447

[103] Sun Z Yu T Jiao Y He D Wu J Duan W. Expression profiles and ontology analysis of circular RNAs in a mouse model of myocardial ischemia/reperfusion injury. Biomed Res Int. (2020) 2020:2346369. 10.1155/2020/2346369 32596283 PMC7273412

[104] Si X Zheng H Wei G Li M Li W Wang H. circRNA Hipk3 induces cardiac regeneration after myocardial infarction in mice by binding to Notch1 and miR-133a. Mol Ther Nucleic Acids. (2020) 21:636–55. 10.1016/j.omtn.2020.06.024 32736292 PMC7393325

[105] Sun JY Shi Y Cai XY Liu J. Potential diagnostic and therapeutic value of circular RNAs in cardiovascular diseases. Cell Signal. (2020) 71:109604. 10.1016/j.cellsig.2020.109604 32201331

[106] Chen JX Hua L Zhao CH Jia QW Zhang J Yuan JX. Quantitative proteomics reveals the regulatory networks of circular RNA BTBD7_hsa_circ_0000563 in human coronary artery. J Clin Lab Anal. (2020) 34:e23495. 10.1002/jcla.23495 32710445 PMC7676214

[107] Ming-Hui Yang HW Sheng-Na Han Xin J.ia Si hang Fei-Fei D. ai Meng-Jiao Z hou Zhongnan. Circular RNA expression in isoproterenol hydrochloride-induced cardiac hypertrophy. Aging. (2020) 12:2530–44. 10.18632/aging.102761 32023551 PMC7041747

[108] Wang K Long B Liu F Wang JX Liu CY Zhao B. A circular RNA protects the heart from pathological hypertrophy and heart failure by targeting miR-223. Eur Heart J. (2016) 37:2602–11. 10.1093/eurheartj/ehv713 26802132

[109] Liu X Wang M Li Q Liu W Jiang H. CircRNA ACAP2 Induces Myocardial Apoptosis After Myocardial Infarction by Sponging miR-29. Minerva Medica. (2020). 32406223 10.23736/S0026-4806.20.06600-8

[110] Li F Long TY Bi SS Sheikh SA Zhang CL. circPAN3 exerts a profibrotic role via sponging miR-221 through FoxO3/ATG7-activated autophagy in a rat model of myocardial infarction. Life Sci. (2020) 257:118015. 10.1016/j.lfs.2020.118015 32629000

[111] Zhou B Yu JW. A novel identified circular RNA. circRNA_010567, promotes myocardial fibrosis via suppressing miR-141 by targeting TGF-beta1. Biochem Biophys Res Commun. (2017) 487:769–75. 10.1016/j.bbrc.2017.04.044 28412345

[112] Tang CM Zhang M Huang L Hu ZQ Zhu JN Xiao Z. CircRNA_000203 enhances the expression of fibrosis-associated genes by derepressing targets of miR-26b-5p, Col1a2 and CTGF, in cardiac fibroblasts. Sci Rep. (2017) 7:40342. 10.1038/srep40342 28079129 PMC5228128

[113] Zhu Y Pan W Yang T Meng X Jiang Z Tao L. Upregulation of circular RNA CircNFIB attenuates cardiac fibrosis by sponging miR-433. Front Genet. (2019) 10:564. 10.3389/fgene.2019.00564 31316543 PMC6611413

[114] Ni H Li W Zhuge Y Xu S Wang Y Chen Y. Inhibition of circHIPK3 prevents angiotensin II-induced cardiac fibrosis by sponging miR-29b-3p. Int J Cardiol. (2019) 292:188–96. 10.1016/j.ijcard.2019.04.006 30967276

[115] Zhang N Wang X. Circular RNA ITCH mediates H2O2-induced myocardial cell apoptosis by targeting miR-17-5p via wnt/beta-catenin signalling pathway. Int J Exp Pathol. (2020) 102:22–31. 10.1111/iep.12367 33350543 PMC7839958

[116] Hu X Ma R Cao J Du X Cai X Fan Y. CircSAMD4A aggravates H/R-induced cardiomyocyte apoptosis and inflammatory response by sponging miR-138-5p. J Cell Mol Med. (2020). 10.1111/jcmm.16093 33219594 PMC8918413

[117] Ji X Ding W Xu T Zheng X Zhang J Liu M. MicroRNA-31-5p attenuates doxorubicin-induced cardiotoxicity via quaking and circular RNA Pan3. J Mol Cell Cardiol. (2020) 140:56–67. 10.1016/j.yjmcc.2020.02.009 32135167

[118] Zhang CL Long TY Bi SS Sheikh SA Li F. CircPAN3 ameliorates myocardial ischaemia/reperfusion injury by targeting miR-421/Pink1 axis-mediated autophagy suppression. Lab Invest. (2021) 101:89–103. 10.1038/s41374-020-00483-4 32929177

[119] Chen L Luo W Zhang W Chu H Wang J Dai X. circDLPAG4/HECTD1 mediates ischaemia/reperfusion injury in endothelial cells via ER stress. RNA Biol. (2020) 17:240–53. 10.1080/15476286.2019.1676114 31607223 PMC6973311

[120] Zhu Y Zou C Jia Y Zhang H Ma X Zhang J. Knockdown of circular RNA circMAT2B reduces oxygen-glucose deprivation-induced inflammatory injury in H9c2 cells through up-regulating miR-133. Cell Cycle. (2020) 19:2622–30. 10.1080/15384101.2020.1814025 32897801 PMC7644149

[121] Cui X Dong Y Li M Wang X Jiang M Yang W. A circular RNA from NFIX facilitates oxidative stress-induced H9c2 cells apoptosis. In Vitro Cell Dev Biol Anim. (2020) 56:715–22. 10.1007/s11626-020-00476-z 33067659

[122] Jin P Li LH Shi Y Hu NB. Salidroside inhibits apoptosis and autophagy of cardiomyocyte by regulation of circular RNA hsa_circ_0000064 in cardiac ischemia-reperfusion injury. Gene. (2021) 767:145075. 10.1016/j.gene.2020.145075 32858179

[123] Gao WQ Hu XM Zhang Q Yang L Lv XZ Chen S. (2021). Downregulation of circFASTKD1 ameliorates myocardial infarction by promoting angiogenesis. Aging. 13:3588–604. 10.18632/aging.202305 33411690 PMC7906207

[124] Zhang Q Sun W Han J Cheng S Yu P Shen L. The circular RNA hsa_circ_0007623 acts as a sponge of microRNA-297 and promotes cardiac repair. Biochem Biophys Res Commun. (2020) 523:993–1000. 10.1016/j.bbrc.2019.12.116 31973814

